# Adipose-Derived Stem Cells in Bone Tissue Engineering: Useful Tools with New Applications

**DOI:** 10.1155/2019/3673857

**Published:** 2019-11-06

**Authors:** Gabriele Storti, Maria Giovanna Scioli, Bong-Sung Kim, Augusto Orlandi, Valerio Cervelli

**Affiliations:** ^1^Plastic and Reconstructive Surgery, Department of Surgical Sciences, University of Rome - "Tor Vergata", Italy; ^2^Anatomy Pathology Institute, Department of Biomedicine and Prevention, University of Rome - "Tor Vergata", Italy; ^3^Division of Plastic Surgery and Hand Surgery, University Hospital Zurich, Switzerland

## Abstract

Adipose stem cells (ASCs) are a crucial element in bone tissue engineering (BTE). They are easy to harvest and isolate, and they are available in significative quantities, thus offering a feasible and valid alternative to other sources of mesenchymal stem cells (MSCs), like bone marrow. Together with an advantageous proliferative and differentiative profile, they also offer a high paracrine activity through the secretion of several bioactive molecules (such as growth factors and miRNAs) via a sustained exosomal release which can exert efficient conditioning on the surrounding microenvironment. BTE relies on three key elements: (1) scaffold, (2) osteoprogenitor cells, and (3) bioactive factors. These elements have been thoroughly investigated over the years. The use of ASCs has offered significative new advancements in the efficacy of each of these elements. Notably, the phenotypic study of ASCs allowed discovering cell subpopulations, which have enhanced osteogenic and vasculogenic capacity. ASCs favored a better vascularization and integration of the scaffolds, while improvements in scaffolds' materials and design tried to exploit the osteogenic features of ASCs, thus reducing the need for external bioactive factors. At the same time, ASCs proved to be an incredible source of bioactive, proosteogenic factors that are released through their abundant exosome secretion. ASC exosomes can exert significant paracrine effects in the surroundings, even in the absence of the primary cells. These paracrine signals recruit progenitor cells from the host tissues and enhance regeneration. In this review, we will focus on the recent discoveries which have involved the use of ASCs in BTE. In particular, we are going to analyze the different ASCs' subpopulations, the interaction between ASCs and scaffolds, and the bioactive factors which are secreted by ASCs or can induce their osteogenic commitment. All these advancements are ultimately intended for a faster translational and clinical application of BTE.

## 1. Introduction

Bone is a complex tissue and participates into several physiological processes, including body movements, mineral (calcium and phosphate) homeostasis and storage, endocrine functions, and, in the bone marrow, hematopoiesis [1, 2]. Bone fractures are among the most frequent organ injuries, and high energy traumas can ultimately result in complex fracture or losses of bone tissue. Additionally, oncological skeletal surgery, malformations, prosthesis revision, or osteomyelitis can determine segmental loss of osseous structures. Usually, bone tissues present an excellent self-repair and regeneration capacity through the recruitment of osteoprogenitor cells from the surroundings, which entails scarless healing as the outcome [3]. Unfortunately, sometimes the damage exceeds bone self-healing capacity ultimately leading to delayed healing, scar formation, and nonunion or persistent bone defects, in the worst scenario [4]. Usually, a scarce or compromised vascularization and a reduced number of progenitor cells underlie these conditions, possibly worsened by patients' comorbidities, lifestyle, or genetic factors [5].

Hitherto, the gold standard to treat these conditions is an autologous bone graft, which is highly biocompatible and has a low risk for rejection. Nonetheless, many drawbacks could hinder good results for autograft, including their limited accessibility, a limited amount of material available, and morbidity at the donor site [6, 7]. To overcome these limitations, bone tissue engineering (BTE) is aimed at recreating bone substitutes that are readily available, highly biocompatible, and with a significant regenerative potential [8]. Isolation and characterization of progenitor cells for therapeutic use have significantly improved the possibilities of BTE [9]. Among staminal elements, mesenchymal stem cells (MSCs), first isolated from bone marrow, offered convenient features since they are obtained from adult patients and have the ability to undergo osteogenic differentiation [10]. More recently, adipose tissue emerged as an optimal source of MSCs [11]. Adipose stem cells (ASCs) exhibit several advantages, even in comparison to bone marrow MSCs. They are easy to harvest and to isolate; they have a high proliferative capacity and differentiative capacity, both toward angiogenic and osteogenic lineages [12].

Although the first successful experiences of BTE using ASCs date back to more than ten years ago [13, 14], a better understanding of different ASC subpopulations, their physiology, differentiative mechanisms, and paracrine actions has allowed a continuous development of new applications for ASC use in designing tissue engineering products (TEPs) [15]. Usually, TEPs combine three factors: (1) scaffold, (2) osteoprogenitor cells, and (3) bioactive factors. The maximization of the osteogenic potential of ASCs led to the development of novel scaffolds. Concurrently, subpopulations of ASCs with an enhanced capacity to induce bone formation have been isolated. The paracrine role of ASCs has also been studied further. On this behalf, ASCs, as an essential source of bioactive factors, can have a high impact also on tissues of the recipient site. In particular, ASC-derived exosomes and microRNAs (miRNAs) have shown significant osteoinductive capacities, thus being another mechanism for ASC-promoted bone formation. In this review, we are going to discuss recent discoveries involving ASC use in tissue engineering, focusing on their role and interactions with the other components of TEPs.

## 2. Adipose Stem Cells (ASCs) and Their Subpopulations

Among MSCs, ASCs present some advantages for tissue engineering applications. Adipose tissue is easy to harvest and contains a higher number of staminal precursors, up to 2,500-fold higher than in bone tissue [16]. However, ASCs share several features with other MSCs, which are defined by a position statement of the International Society for Cellular Therapy (ISCT). Three minimal criteria define cultured MSCs: (i) plastic adherence in standard culture conditions; (ii) positivity for the expression of CD105, CD73, and CD90 and negativity for CD45, CD34, CD14 or CD11b, CD79*α* or CD19, and HLA-DR surface molecules; and (iii) potential to undergo trilineage differentiation (adipogenic, chondrogenic, and osteogenic) [17]. Nonetheless, it has emerged over time that, among ASCs, different subpopulations could be identified. ASCs are part of the perivascular niche, but the lack of expression of CD31 distinguishes them from endothelial cells that are positive for it [18].

CD146, an adhesion molecule also known as Mel-CAM (melanoma cell adhesion molecule), has been used for the identification and purification of perivascular progenitor cells [19]. Among the perivascular ASCs, two different subpopulations could be identified using the combined analysis of different surface markers, like CD146, CD34, and CD31: (i) cells which are CD146+ CD34- CD31- also defined as pericytes and (ii) CD146- CD34+ CD31- also defined as adventitial cells [20–23]. Adventitial cells are located in an outer layer of the supra-adventitial fat, whereas pericytes are closely associated with the microvasculature [23]. They hold strict relationships with endothelial cells, and their interplay has a significant role in the regulation of angiogenesis. [24]. Some hypotheses consider pericytes as a more staminal form that undergo a differentiation process from inside outwards [25]. Both cell types share with MSC features like growth, morphology, surface markers, and clonal multilineage differentiation potential [26–29]. According to Rad et al., the buccal fat pad, compared to abdominal and hip fat, showed the highest amount of CD146-positive cells, higher proliferation rate, and expression of osteogenic and angiogenic markers [30].

Many authors investigated the osteogenic capacity of sorted CD146+ CD34- CD31- pericytes from adipose tissue and evaluated their possible use for bone tissue engineering. James et al. demonstrated for the first time the higher osteogenic capacity of sorted pericytes from human lipoaspirate, compared to matched unsorted ASCs, both in vitro and in vivo [31]. Pericytes had a better osseous differentiation compared to unsorted cells, under osteogenic conditions in vitro. CD146+ cells formed more bone than unsorted cells in vivo as well, even without predifferentiation. Pericytes confirmed a high osteogenic capacity when seeded on a cancellous bone scaffold and tested on a calvarial critical-sized defect [32]. In a rat spine fusion model, human pericytes were able to induce both intramembranous and endochondral bone formation. A complete fusion of lumbar segments was obtained in all the rats treated with pericytes and ossification, bone deposition and bone strength increased in a dose-dependent fashion [33]. Efficacy of human pericytes for bone formation was also evident in an atrophic bone nonunion animal model. An increased fracture callus size and increased mineralization after three weeks finally resulted in increased bone union [34]. Interestingly, in all these xenografts models, aside from the osteogenic differentiation of human pericytes, a paracrine effect was evident, which determined a repopulation of the defect by host cells. Over time, only a little chimerism could be detected, with a limited presence of human cells. Whether the trophic/secretory effect is more critical for new bone formation than the cellular osteogenic differentiation is not clear yet [35].

Wang et al. [36] have demonstrated that cocultivation of CD146+ pericytes and patient-matched CD34+ adventitial cells resulted in a better osteogenic and vasculogenic differentiation. When evaluated on a critical-sized calvarial defect model in NOD/SCID mice, the combination of CD146+ pericytes with CD34+ adventitial cells determined a more efficient reossification than either cell type alone. It could be inferred that CD146+ pericytes and CD34+ adventitial cells display overlapping and complementary roles, even though with different functions in bone defect repair. Consequently, CD146+ pericytes and CD34+ adventitial cells may demonstrate a synergistic effect on bone healing when applied together as a combined therapy.

Therefore, according to literature data, a more precise selection and expansion of cellular subpopulations, under Good Manufacturing Practice (GMP) conditions, could ultimately lead to more efficient engineering protocols.

## 3. Scaffolds and Adipose Stem Cells

Scaffolds are three-dimensional constructs that are designed and intended to recreate the extracellular matrix (ECM), thus promoting the regeneration of a functional bone. Scaffolds should guide the healing process, promote the differentiation of progenitor cells, and mimic the extracellular environment while providing mechanical support [37].

Various scaffolds have been investigated for bone tissue engineering (BTE) and synthesized using both inorganic and organic materials [38]. Generally, an ideal scaffold should have precise features in order to have an optimal integration and provide the correct stability [39]. An ideal scaffold should be biocompatible, thus eliciting a minimal inflammatory and immunological response, and it should be biodegradable, which means that it should be substituted entirely, over time, by autologous tissue. The ability to mimic the ECM, to facilitate hydroxyapatite (HA) formation and mineral deposition, and ultimately to provide a physical structure suitable for bone growth inside and across it is defined as osteoconduction. Even though stainless steel too has been proven to be osteoconductive, biodegradable scaffolds offer the best osteoconductive properties [40]. Nonetheless, a controlled scaffold degradation is desirable in order to allow the new tissues to grow into it while the scaffold guarantees adequate mechanical support and stiffness until the process of integration ends. This necessity is particularly true in load-bearing areas [41].

Under this aspect, it is fundamental to balance carefully two properties in a scaffold: its porosity and its stiffness. The first allows an optimal vascular inosculation and cellular ingrowth, mimicking the native trabecular bone, while the second is necessary for adequate structural support, which is less in a highly porous material [39].

Porosity is a critical parameter for the interactions between cells and the scaffold. A too narrow diameter of the pores can limit the penetration throughout the scaffold of newly formed vascular structures and cell migration, while too wide pores impair the surface area available for cell adhesion [42, 43]. Both these cellular processes are critical for cell differentiation, proliferation, and migration, ultimately determining an optimal integration of the scaffold and its suitability for BTE [44]. Therefore, optimization of pore size is fundamental during scaffold construction [45].

As mentioned above, osteoconductive scaffolds exert their properties passively and act as relatively inert support that guides bone ECM formation, cell migration, and proliferation toward the regeneration of the defect. Osteoconductive materials are efficacious mainly with partly differentiated cells like osteoblast and preosteoblast but do not induce osteogenic differentiation of osseous progenitor cells and mesenchymal stem cells (including ASC) [8]. On the other hand, osteoinductive biomaterials can recruit progenitor cells and stimulate their osteoblastic commitment and differentiation, which allows de novo bone formation [46, 47]. Autografts and tissue engineering products (TEPs), where scaffolds are enriched with stem cells or osteogenic factors, e.g., BMPs [48, 49], usually fall among osteoinductive materials. However, some biomaterials, like some calcium phosphate cements, have intrinsic osteoinductive features even without the addition of osteogenic factors [50, 51]. Osteoinduction, a crucial characteristic of scaffolds, has been widely exploited together with ASC and osteogenic factors in order to obtain optimal TEPs. Alongside with excellent osteoinductive properties, a full osteointegration of the scaffold into the host bone relies also on adequate vascular support. Neoangiogenesis into the graft should allow connections to the host microvasculature. A stable vascularization of the graft and a vascular supply to the central part of the graft can hold its bioactive function and avoid necrosis, which is a significant risk to be considered especially in large bone grafts [52, 53]. The capacity of a biomaterial to host vascular ingrowth is named angioconduction, whereas the ability of a biomaterial to actively stimulate and promote the formation of new vessels goes under the name of angioinduction [39]. Osteoinduction and osteoconduction, angioinduction and angioconduction together with biocompatibility, biodegradability, and physical and mechanical properties of the scaffold are crucial in order to obtain ideal TEPs for bone reconstruction.

ASCs have been employed for bone reconstruction together with several scaffold types derived both from inorganic and organic sources. Several materials have been investigated for scaffold production, ranging from decellularized tissue matrices to inorganic ceramics (e.g., hydroxyapatite (HA), coralline-derived hydroxyapatite (cHA), tricalcium phosphate (TCP), calcium sulphates, glass ceramics, calcium phosphate-based cements, and bioglass), synthetic biodegradable polymers such as polylactic acid (PLA) and polyglycolic acid (PGA), or combinations of two or more of them [54].

ASCs have been tested on several biomimetic scaffolds in order to obtain an optimal osteogenic differentiation and ultimately an ideal TEP for bone reconstruction. The capacity of a scaffold to directly induce osteogenic differentiation of ASCs, without bioactive factors, could help in simplifying the approach to bone reconstruction in the future.  Table 1 provides a synthetic overview of recent literature about the combination of scaffolds and ASCs for bone tissue engineering.

### 3.1. Decellularized Matrices

Scaffolds derived from acellular matrices have several advantages. They have a structure similar to that of the original bone extracellular matrix; they can recapitulate the complex microenvironment of naïve bone, and they have shown significative osteoinductive capacities [74]. These materials require a donor tissue and present the possible risk of transmission of infectious diseases as the main drawback [75].

ASCs have been used together with acellular matrices from different sources. Allografts and xenografts have been derived mainly from bone tissue [55, 58, 60, 76, 77] but also from small intestine submucosa [57] or from adipose tissue [59].

Ko et al. tested a nanostructured tendon-derived scaffold on a mouse model of calvarial critical-sized bone defects together with human ASCs (hASCs). hASCs seeded on this nanostructured tendon-derived scaffold had an enhanced focal adhesion and an increased osteogenic differentiation after 21 days. The same construct was layered on the mouse calvarial defects, ultimately obtaining well-vascularized bone tissue and a defect closure in 8 weeks [56].

Liu et al. [58] combined hADSC with a deproteinized bone matrix, derived from New Zealand rabbits. The heterogeneous deproteinized bone (HDB) has no severe immunogenicity, and it holds a natural porosity adequate for cell adhesion proliferation and differentiation [78]. Composites with HDB were obtained both with naïve hASCs and with hADSC which have already undergone a partial osteogenic differentiation. The constructs were tested on a murine 4 mm-long radial bone defect. Both types of HDB-ASC composites showed a strong osteogenic ability when compared to control groups, at four and eight weeks. The best performance was obtained by the HDB-ASC constructs combined with osteogenic ASCs which filled the bone defect area and were practically indistinguishable from the host bone tissue.

Porcine small intestine submucosa (SIS) was also investigated as another option for bone tissue engineering in a mouse calvarial defect model [57]. The SIS scaffold was initially seeded with osteoblasts from the MC3T3-E1 cellular line in order to obtain ECM deposition. After four weeks, the ECM-SIS scaffold was decellularized. hASCs seeded on the ECM-SIS scaffold underwent osteogenic differentiation even in the absence of the osteogenic differentiation medium. They were significantly more efficient in bone tissue formation when compared to those seeded in the SIS-only scaffold. The murine model confirmed these in vitro data. In mice, constructs made of ECM-SIS plus hADSCs had the best performance in comparison to scaffolds alone and also to ADSCs seeded on SIS-only scaffolds.

Among human allogenic materials, Wagner et al. [60] tested a construct made of decellularized human cancellous bone seeded with hASCs, both in vitro and in a murine femur model with a critical-sized defect. The scaffold was able to increase the osteogenic differentiation of hASCs when compared to controls. In the murine model, after four weeks, seeded scaffolds showed a significative higher formation of vital bone in comparison to unseeded controls Moreover, the scaffold showed optimal osteoconductive properties and favored both the differentiation of hASCs into CD31+ endothelial cells and an increased neoangiogenesis.

In a study by Vériter et al. [55], an ASC-based product was created combining ASCs and human demineralized bone matrix (DBM) which has powerful osteoinductive properties.

ASCs at passage four were incubated with an osteogenic differentiation medium for 15 to 18 days and then added to DBM. These grafts were implanted on 11 patients with bone nonunion from different etiologies, including postoncological reconstructions and congenital defects. No serious adverse events or oncological recurrences were reported during the follow-up of 54 months. The grafts were fully integrated, determining the healing of the bone nonunions.

Adipose tissue was also reported as a potential candidate for a scaffold intended for bone reconstruction. Guerrero et al. [59] cultivated human adipose tissue from liposuction for three weeks in a proliferation medium on agarose-coated plates in order to obtain a construct called Adiscaf. Adiscaf was confronted with a collagen scaffold (Ultrafoam) seeded with a monolayer of ASCs from the same patient. Both Adiscaf and the collagen construct were held for four weeks in a chondrogenic differentiation medium. Adiscaf differentiated into cartilage tissue with the synthesis of glycosaminoglycans and type II collagen. When implanted into a subcutaneous pouch on nude mice, the cartilage tissue formed into the Adiscaf was able, after eight weeks, to produce a higher amount of mineralized tissue compared to Ultrafoam-based constructs. The ectopic bone formed through endochondral ossification, and Adiscaf was superior to Ultrafoam both in vitro and in vivo, thus being a possible candidate for the generation of osteogenic grafts for bone repair.

### 3.2. Ceramics

Several synthetic materials, derived both from inorganic and organic origins, have been tested together with ASCs as scaffolds for bone tissue engineering.

Calcium phosphate ceramics (e.g., hydroxyapatite (HA), coralline-derived hydroxyapatite (cHA), tricalcium phosphate (TCP), calcium sulfates, glass ceramics, calcium phosphate-based cements, and bioglass) have been introduced about 40 years ago as bone substitutes [54, 79]. They have excellent osteoinductive properties [80, 81], and they have been used alone to treat distal radial fracture [82, 83], but they have the relative disadvantage of a high brittleness, TCP in particular [84]. Therefore, it could be problematic to use ceramics, especially when a construct for load-bearing areas is needed [85]. The combination of calcium ceramics together (e.g., HA and TCP) or mineral substitution with strontium or magnesium can improve their mechanical properties, their biodegradability, and their osteoinductive capacity [86]. The association of *β*-TCP and HA has better mechanical properties than *β*-TCP alone, while it also enables a faster and higher bone ingrowth rate than using HA alone [87].

It was demonstrated that the extracellular calcium concentration could influence the differentiation of ASCs toward an osteogenic phenotype also without any other soluble osteogenic factor [88]. Elevated calcium induced osteogenesis and inhibited chondrogenesis in hASC even in the presence of the chondrogenic differentiation medium. This phenomenon could be an explanation for the osteoinductive and osteoconductive capacities of calcium phosphate ceramics like tricalcium phosphate (TCP) [66]. Interestingly, based on these observations, Mellor et al. developed a stacked polylactic acid (PLA) nanofibrous scaffolds containing either 0% or 20% tricalcium phosphate (TCP) nanoparticles. In chondrogenic differentiation mediums, different extracellular calcium concentrations, in different layers of the scaffold, determined ASCs' commitment either toward osteogenesis or into chondrogenesis, thus exploiting the different calcium concentrations for site-specific differentiation.

In an in vitro model, an HA/TCP scaffold was able to enhance the osteogenic differentiation of hASCs, more than doubling the cellular alkaline phosphatase activity when added to the osteogenic differentiation medium [61].

*β*-TCP with ASCs was tested in several clinical settings, particularly in maxillofacial surgery and neurosurgery, with excellent results in terms of ossification [62, 89–91].

In a phase I study by Farré-Guasch et al. [62], ten patients undergoing maxillary sinus floor elevation (MSFE) for dental implant placement were divided into two groups. Each group received in a single-step procedure autologous stromal vascular fraction (SVF), containing ASCs, mixed either with *β*-TCP or with biphasic calcium phosphate (BCP), consisting of 60% hydroxyapatite (HA) and 40% *β*-tricalcium phosphate (*β*-TCP). A control group was treated with ceramics only. Both the study groups, compared to the control group, had an increased vascularization of the implanted area, which ultimately determined an enhanced bone formation.

cHA was tested by a Chinese group for the construction of a vascularized tissue-engineered bone together with a double-cell sheet complex [63]. A double-cell sheet (DCS) was created inducing ASCs toward vascular and osteogenic commitment at the same time. The DCS was engineered with cHA, using different patterns. The pattern organized with endothelial cell sheets covered with osteogenic cell sheets had the best results. Moreover, the DCS-cHA complexes had, in general, better bone maturation and vascularization of the graft compared to DCS or cHA alone, when implanted in nude mice and tested for ectopic ossification.

As mentioned above, incorporating minerals, like the strontium (Sr), into ceramics like HA, could enhance the osteoinductive properties of the inorganic cements. Sr ions have the ability to regulate osteoclast activity [92] and to improve the osteointegration when incorporated into bioactive scaffolds [93] or together with HA [94]. ASCs engineered in a strontium hydroxyapatite (SrHA) scaffold were used as a tissue-engineered construct (cSrHA) on a sheep model of osteoporosis [64]. ASCs demonstrated to act synergically with Sr ions, thus enhancing the osteogenic capacity of the SrHA scaffold when compared to acellular scaffold controls. ASCs adhered and proliferated on the SrHA scaffold, retaining an optimal osteogenic capacity in vitro. In the in vivo model, the osteointegration of the construct was superior to controls.

### 3.3. Synthetic Polymers and Hybrid Scaffolds

Synthetic polymers have been extensively investigated because of many advantages that they could offer for scaffold design, including biocompatibility, controllable biodegradability, and their physio/chemical properties. Polylactic acid (PLA), polyglycolic acid (PGA), polycaprolactone (PCL), and the copolymer poly(lactic acid-co-glycolic acid) (PLGA) are among the most frequently tested for bone tissue engineering [95]. ASCs have been combined with synthetic polymers with excellent support to osteogenic differentiation in several preclinical models [1, 96–98]. Synthetic polymers offer advantageous chemical, biological, and flexible mechanical properties. They are highly pure, readily reproducible, and easy to be tailored to fulfill specific needs. Nonetheless, synthetic polymers have several drawbacks when used alone. They have a fast degradation rate and a reduced compressive modulus, and they lack osteoinductive capacity, even though PCL has shown the ability to increase the osteogenic differentiation of various human tissue-derived mesenchymal stem cells (including ASCs) through the activation of the Wnt/*β*-catenin and Smad3 signaling pathways [99]. Moreover, some synthetic polymers, such as PLGA and poly-L-lactic acid (PLLA), degrade into nonbiocompatible products, often acids, which can lead to cell dysfunction or death, via perturbation of the scaffold microenvironment. An increased acidity of the tissue microenvironment, determined by high concentrations of these degradation products, could also result in adverse responses, such as inflammation or fibrous encapsulation [100, 101]. For the sake of overcoming their drawbacks, synthetic polymers have been often used over the years in combination with other materials like ceramics (e.g., HA or TCP), collagen, or natural polymers into hybrid scaffolds [75]. Hybridization with other materials allows synthetic polymers to increase their resistance to compression and their osteoinductive capacity and to prolong their degradation time. At the same time, the high versatility provided by synthetic polymers allows using these materials for scaffold creation through several bioengineering techniques like 3D bioprinting or electrospinning [37, 102]. Furthermore, synthetic polymers (e.g., PLGA) can be used for encapsulation and progressive release of bioactive molecules, exploiting the biodegradability profile of the materials [103–105].

Lee et al. [67] tested a 3D-printed PCL/TCP scaffold seeded with ASCs on a canine model of a maxillary bone defect. This scaffold enhanced the osteogenic capacity of ASCs as it was demonstrated by RT-PCR and Western blot analysis for COL1, OCN, and RUNX2. Moreover, 3D CT scans demonstrated a process of ossification of the defect after 12 weeks, confirmed by the histological analysis.

ASCs were able to undergo direct osteogenic differentiation induced by different polymer-mineral constructs in a murine model. In a study by Duan et al. [68], the different scaffolds, made of synthetic polymers mixed with different ceramics, were able to promote osteogenic differentiation of ASCs in the absence of differentiating factors. Scaffold seeded with ASCs displayed more ECM and osteoid tissue compared to those scaffolds without cells.

The addition of starch to PCL (SPCL) was used to create a wet-spun SPCL scaffold which proved to be biodegradable and biocompatible and able to harbor undifferentiated hASCs and support their proliferation and osteogenic differentiation [65]. Starch increased the resistance to tensile forces of PCL, thus improving its mechanical properties. A construct made of undifferentiated hASCs and the SPCL scaffold was tested on a murine calvarial defect model. ASCs improved the osteogenic function of SPCL and promoted significantly better bone deposition. SPCL was able to induce osteogenic differentiation in ASCs even without the addition of osteogenic factors, and the newly formed tissue was well integrated into the surrounding tissues.

### 3.4. Natural Polymers

Natural polymers are derived from various sources, mainly animals and plants [106]. They were among the first scaffolds to be studied in combination with hASCs to design TEPs because of their properties close to those of the ECM [107–111]. Natural polymers combined with hASCs for the use in bone tissue engineering applications are often animal derivatives. However, polysaccharides too like chitosan [112–114] or cornstarch [65, 115] have been investigated for this purpose. In between the animal-derived polymers, ASCs have demonstrated to positively interact mainly with fibrin [116], collagen [117, 118], gelatin [111, 119, 120], and silk [69, 121, 122], through the recognition of specific domains present in polymers' structures. These polymers have a good biodegradability profile, and they can be degraded, modified, or adsorbed by the action of naturally occurring enzymes [123]. Since they are versatile, they are often used in combination with other scaffolding materials (e.g., ceramics or synthetic polymers), resulting in new constructs that incorporate their biodegradability and their biological properties, which mimic those of the ECM but have superior mechanical characteristics [124]. These composite constructs are easy to use and to adapt to multiple tissue engineering techniques like phase separation, electrospinning, or 3D printing [125–129].

When seeded on composite scaffolds synthesized with natural polymers, ASCs proved optimal adhesion, proliferation, and osteogenic differentiation, in many cases without the use of differentiating agents, both in vivo and in vitro.

The combined use of collagen and HA proved optimal osteoinductive and osteoconductive properties both in vitro and in vivo [70, 71, 130, 131].

Mazzoni et al. [71] proved that the collagen/HA is an ideal microenvironment for hASC adhesion and proliferation, and it determines an upregulation of osteogenic genes and an improvement of cellular viability and matrix mineralization, similar to that obtained under osteogenic culture conditions.

It has been demonstrated that collagen and HA scaffolds activate distinct osteogenesis signaling pathways in ASCs [130]. Collagen seemed to stimulate ECM deposition and osteoblastic differentiation through the stimulation of the extracellular signal-regulated protein kinase (ERK) pathway, whereas HA stimulated the osteogenic differentiation of ASCs via the Wnt/*β*-catenin pathways which determined an increase in the osteoprotegerin (OPG)/receptor activator of nuclear factor-kappa *β* ligand (RANKL) ratio. The upregulation of these two pathways could explain the synergistic effects of collagen and HA on the osteogenic differentiation of ASCs.

Calabrese et al. [70] tested the biocompatibility and the osteogenic capacity of hASCs on collagen-HA scaffolds in vitro. Undifferentiated ASCs were able to undergo full differentiation into mature osteoblasts even without the addition of an osteogenic medium, thus demonstrating a per se osteoinductive capacity of the scaffold, which could be exploited in order to have more straightforward translational applications in the future.

Collagen has also been used together with synthetic polymers like PGA. ASCs were seeded in combination with a construct made of collagen sponge and PGA on a rabbit critical-sized calvarial bone defect [72]. In this animal model, the scaffold itself was able to promote the healing of the defect and no difference was noted in between the scaffold-only group and the scaffold+ASC group.

Silk fibroin is a valuable alternative to other natural polymers for TEPs in combination with ASCs. It presents several advantages: in its spongeous form, it has a high mechanical and tensile strength and it has a high porosity with the possibility of different pore sizes, which eases cell attachment, proliferation, and the development of a supportive vascular network. It was demonstrated that a pore dimension of 400–600 *μ*m determines the best bone tissue formation outcomes, as evidenced by the enhanced production of bone protein (osteopontin, collagen type I, and bone sialoprotein) and calcium deposition, and the increased total bone volume, in a porous HFIP-derived silk fibroin scaffold seeded with hASCs. In an osteogenic differentiation medium, the osteogenic performance in term of alkaline phosphatase activity (AP) at week 2 and new calcium deposition at week 7 was comparable to those of cells cultured on a decellularized trabecular bone [69].

A Korean group used electrospun silk fibroin nanofiber scaffolds, which were functionalized with two-stage HA particles. HA particles were immobilized via polydopamine-mediated adhesive chemistry. ASCs' interactions with this construct were tested both in vitro (under standard and osteogenic culture conditions) and in vivo, on a murine critical-sized calvarial bone defect model [56]. Furthermore, they also tested hASCs transfected with the TAZ gene. TAZ is a transcriptional modulator that triggers the osteogenic differentiation of ASCs. Constructs seeded with TAZ-transfected ASCs had the best osteogenic performance both in vitro and in vivo. However, those seeded with wild-type hASCs also proved to be superior to the unseeded scaffold. This study highlighted the possible future utility of silk fibroin nanofibrous scaffolds, enriched with inorganic components and used in combination with ASCs for bone tissue engineering.

## 4. Bioactive Factors

Bioactive factors are an integrating part of bone tissue engineering strategies, and they usually have osteoinductive and angiogenic properties [102].

Several factors like growth factors (like those in the platelet-rich plasma) or the bone morphogenetic proteins (BMPs) or drugs like simvastatin or RNA products like miRNAs have demonstrated the ability to induce osteogenic differentiation and angiogenesis into ASCs and in the host tissues [132–137].

Parallelly, the paracrine action of ASCs has become more and more evident over the years and it is responsible for their therapeutic effects, together with their differentiation capacity [138]. These paracrine effects are mostly attributable to soluble factors and exosomes which control regeneration processes and the repair of damaged sites by modulating migration, proliferation, and differentiation [139]. Soluble factors and exosomes, with their “cargos,” are promising options for bone tissue engineering, both improving ASCs' osteogenic differentiation and enhancing bone formation through a paracrine osteoinductive activity.

Several studies have recently demonstrated that ASCs-derived exosomes could have biological effects close to those of the proper cellular component, in bone regeneration, as well as in neoangiogenesis and wound healing [140–142].

### 4.1. Growth Factors (GFs)

Usually, the ECM stores many of these bioactive factors, including many growth factors (GFs), such as FGF, TGF*β*, BMPs, VEGF, and IGF I and II. In specific pathophysiological conditions, these GFs are released from the ECM and become available for cells [143]. When administered exogenously for therapeutic purposes, these factors have short half-lives in their natural form. Without any protection, they are readily biodegradable and their elimination through the bloodstream rapidly lowers their local concentrations. Lower concentrations reduce their efficacy into the target organs, while systemic diffusion increases the risk of adverse effects [38].

Therefore, more adequate delivery strategies should be investigated and employed in order to have stable and efficacious steady-state levels and a prolonged release over time, which mimics what actually happens under physiological conditions. Embedding bioactive factors and exosomes into scaffolds, microsphere encapsulation, and enhanced expression through gene transfection into mesenchymal cells are among the most investigated options [39, 128, 140, 144–148].

As mentioned above, efficacious strategies in bone tissue engineering rely both on an optimal ossification of the constructs and on its adequate vascularization, which allows integration and avoids total or partial necrosis of the graft. For these purposes, GFs have been extensively studied, and among them PDGF-BB, the TGF-*β* family (that includes BMP proteins), FGFs, insulin-like growth factors, and VEGF. In particular, VEGF and BMP2 were considered as the principal actors in the bone repairing process, respectively, on the vascular and osteogenic side [149]. It has been demonstrated that VEGF and BMP2 act synergistically in favor of bone formation and their coadministration is more efficacious than BMP-2 alone [150]. Three BMPs have already been approved in clinics: BMP-2 (Infuse bone graft) since 2003, BMP-7 (OP-1 putty) (from 2003 to 2014 when it was withdrawn), and rhPGDF-BB (Augment® bone graft), since 2015 [9]. BMP-2 has already been used in many clinical situations together with ASCs. In several case series, a construct made of hASCs, with BMP2, onto a *β*-TCP scaffold, proved efficacious for the reconstruction of large maxillary or mandibular defects and those of craniomaxillofacial hard tissues in general [91, 151, 152]. Parallelly, resorbable scaffolds seeded with hASCs combined with BMP2 were used to reconstruct large craniofacial bony defects in 20 patients [90].

Many GFs useful in bone regeneration, such as b-FGF or FGF-2, IGF-1, PDGF-BB, and VEGF, are contained in significative quantities into the platelet-rich plasma (PRP) [153]. PRP can be used to induce the osteogenic differentiation of ASCs, both in vitro and in vivo [132, 154]. Several authors incorporated PRP into different types of scaffold in order to have a controlled and prolonged release of GFs and to favor ASC-mediated bone formation [155–158]. These systems allowed an increased osteogenic capacity of ASCs and an enhanced bone deposition, both in vitro and in animal models.

However, ASCs are not only the target of GFs, but they are also able to produce and secrete them in both an autocrine and paracrine way. They release microvesicles (MVs) containing angiogenic factors like FGF2, PDGF, VEGF, MMP2, and MMP9 and osteogenic molecules, such as BMP2 [159, 160]. Furthermore, ASCs can be engineered to express and release osteogenic factors like BMP-2. Using this method, Lin et al. were able to significantly increase calvarial healing through the BMP2-expressing ASCs/gelatin constructs and highlighted the importance of combinations of growth factors and scaffold in healing pathways and their efficacy together [161].

### 4.2. Exosomes and miRNAs

Exosomes are extracellular vesicles with a spheroidal/discoid shape which have a diameter in between 30 and 150 nm. They play a pivotal role in intercellular communication since they show the ability to transport specific molecules like proteins, lipids, DNAs, and RNAs from cell to cell [162, 163]. The precise mechanisms through which exosomes determine the osteogenic differentiation of ASCs is still under debate. It has been discovered that exosomes could regulate the functions of target cells through epigenetic changes, thus determining the promotion of bone tissue repair and also their fate through the induction of proliferation or apoptosis. In these processes, mainly proteins and RNAs are involved with significant roles [164–166].

Exosomes derived from MSCs in general, and in particular those from ASCs, have been investigated in the last years as a possible strategy for osteogenic differentiation, to employ as an alternative to osteogenic differentiation mediums, GFs, and genetic modification [140, 167, 168].

Li et al. [140] tested the efficacy of exosomes derived from osteogenically committed ASCs in promoting the osteogenic differentiation of bone marrow MSCs (bmMSCs) and bone tissue formation. In order to mimic a physiological release, the exosomes were bound to a PLGA/PDA matrix, which allowed a slow and controlled release regulated by its biodegradability properties. In about 48 hours, bmMSCs almost completely internalized the exosomes that stimulated cell proliferation, migration, and osteogenic differentiation, in vitro. Moreover, the cell-free constructs made of PLGA/PDA+exosomes, implanted on a murine critical-sized calvarial bone defect, actively promoted stem cell migration, homing, and new bone formation, in a better way than the PLGA/PDA control scaffold.

In a study by Lu et al. [169], the efficacy of ASCs' exosomes was also confirmed on human primary osteoblastic cells (HOBs), stimulating proliferation, differentiation, and bone-forming capacity. They also demonstrated that preconditioning the ASCs with tumor necrosis factor-alpha (TNF-*α*), for three days, further increased the capacity of ASCs' exosomes to induce osteogenic differentiation. They supposed that the priming with TNF-*α* mimics the acute inflammatory phase following a bone injury. They demonstrated that exosomes, from TNF-*α*-preconditioned ASCs, stimulated the osteogenic gene expression through the Wnt signaling pathway, which is a fundamental pathway in osteogenic differentiation [170, 171].

Yang et al. [172] characterized the exosomes from both osteogenically differentiated and undifferentiated ASCs. In the absence of osteogenic factors, exosomes from osteogenically differentiated cells were able to promote osteogenic differentiation in undifferentiated ASCs, whereas exosomes from undifferentiated ASCs were not. Moreover, ASC-derived exosomes were internalized by target ASCs faster than by other cell types, like bone marrow MSCs (6 h vs. 48 h). According to the authors, this element could be in favor of the combined use in bone tissue engineering of ASCs with ASC-derived exosomes, which limits the loss of exosome, due to a more prolonged uptake, and ultimately leads to a more efficient promotion of bone regeneration. To explain the differences between differentiated and undifferentiated ASCs, they have furthermore analyzed the miRNAs' expression profiles of both cell types. Two hundred thirty-four genes were differently regulated comparing osteogenic ASCs' exosomes to those of undifferentiated ASCs (201 upregulated and 33 downregulated). Most of these miRNAs were related to signaling pathways involved in the osteogenetic process, like the MAPK, the Wnt, and the TGF-*β* signaling pathway. For example, the level of miR-130a-3p was significantly higher in the exosomes from osteogenic ASCs. This miRNA targets and blocks SIRT7, an antagonist on the Wnt pathway, which results ultimately upregulated [173, 174]. The mir-130a-3p/SIRT7/Wnt axis could be a molecular mechanism behind exosomes' efficacy in the regulation of ASC osteogenic differentiation.

As aforementioned, miRNAs play an essential role in the regulation of several biological processes, including osteogenesis, both positively and negatively.

Apart from those contained in ASCs' exosomes, miRNAs can be added into tissue-engineered constructs via different methods in order to improve the osteogenic efficacy of ASCs. miRNAs can be included in the structure of the scaffold which regulates their release, or they can be transfected through viral vectors into ASCs. Viral transfection carries with it, of course, some safety issues, which should be further evaluated, concerning the future translation into clinical practice [146]. An increased expression of miRNAs like miR-148b, miR-26a, miR-135, or miR-130a-3p could enhance the osteogenic processes and increment bone formation [175–179].

Rat ASCs transfected with a lentivirus expressing miR-26a and seeded on an HA scaffold showed an upregulation of proosteogenic genes and an increased bone-forming capacity. This construct, transplanted in a rat tibial defect model, was able to fully close the gap in 12 weeks [180]. mir-148b proved excellent osteogenic capacity and showed a synergistic action together with BMP-2. It was tested on hASCs in multiple in vitro and in vivo models [177–179]. Two studies by Li et al. and Liao et al. transfected undifferentiated hASCS with baculoviruses coexpressing miR148 and BMP-2. mir-148b and BMP-2 were prolongedly overexpressed, and osteogenic differentiation of ASCs was increased in both studies. Similarly, transfected hASCs seeded onto a PLGA scaffold were able to close a murine calvarial bone defect in 12 weeks.

Qureshi et al. [178] combined a truncated miR-148b mimic with photoactivated silver nanoparticles. Undifferentiated hASCs were transfected with the photoactivated miRNA148b-silver nanoparticle conjugates and seeded onto PCL scaffolds. This miRNA delivery system allows to potentially control the differentiation in vivo since miRNA conjugates remain inert until photoexposed at the appropriate dosage and wavelength. At 12 weeks, constructs made of transfected hASCs+PCL scaffold showed a statistically significant better closure of a mouse calvarial bone defect when compared to the control groups.

Conversely, other miRNAs such as miR146a, miR-17, miR-23a, and miR-31 downregulate the BMP2-induced osteogenesis, suppressing BMP-2 and several downstream factors like SMAD1/4, Runx2, and Osx. TGF*β*1 upregulates the expression of these miRNAs. Therefore, antagonists of these miRNA could ultimately determine an improved bone tissue deposition [133, 175, 181]. A lentivirus expressing an antisense miR31 was transfected into rat ASCs that were tested by Deng et al. [181] on a rat critical-sized defect together with a *β*-TCP scaffold. The knockout of miR-31 increased the bone volume and the bone mineral density and decreased the scaffold residue in vivo, thus dramatically improving the repair of critical-sized defects at eight weeks.

Similarly, rat ASCs modified in order to express an anti-miR146 were tested both in vitro and in vivo. The inhibition of miR-146 greatly enhanced ADSC-mediated bone regeneration and bone deposition in the animal model [175].

A synthetic overview of the use of bioactive factors for ASC differentiation is provided in Table 2.

## 5. Conclusions

ASCs confirmed a central role in BTE, providing many new solutions and a high versatility of application, as was evident both in vivo and in vitro. The use of ASCs for regenerative purposes has shown several advantages in comparison to other MSCs, but their interactions with the microenvironment and how these interactions affect their differentiation are still unclear. Different subpopulations of ASCs, such as pericytes and adventitial cells, have demonstrated a better performance in terms of angiogenic and osteogenic differentiation compared to unsorted ASCs. Moreover, an accurate definition of the intrinsic differentiative properties of ASCs' subpopulations could help to find the most appropriate cell for each reconstructive purpose.

New scaffolds, designed to mimic the ECM, thus creating a biological niche to harbor ASCs, are aimed to be fully biocompatible and biodegradable. They should provide adequate mechanical support and promote optimal integration into the host tissues. In this sense, scaffolds could have a strict interaction with ASCs, through the induction of their commitment toward osteogenic lineage and the controlled release of bioactive factors that enhance bone formation and vascular integration of the graft. This proactive role of scaffolds could limit in the future the necessity for predifferentiation of ASCs, which could be committed toward the desired lineage directly in situ. At the same time, bioactive factors, released by the scaffolds, could increase the recruitment and differentiation of progenitor cells from the surroundings. In this sense, ASCs, either naïve or genetically modified, display important paracrine features. ASCs do not contribute to the defect closure via their osteogenic differentiation only. The exosomes and the miRNAs, secreted by osteodifferentiated ASCs, demonstrated to be able to induce the same processes in other cells. Therefore, ASCs are, at the same time, a target and a source of bioactive factors. Fine-tuning of all these components is needed to design constructs that are close to physiological tissues, highly integrable, widely available, and ready to use. The selection of proper elements could help in the future in simplifying the design and the use of TEPs, by reducing the necessity of strong and nonphysiological differentiative stimuli. Tailoring TEPs on specific needs would lead toward easier translational and clinical applications of bioengineering constructs, paving the way for optimal bone reconstructions.

## Figures and Tables

**Table 1:** Recent literature about the combination of scaffolds and ASCs for bone tissue engineering.

Authors and year of publication	Type of cells	Type of scaffold	Experimental model	Results
Decellularized matrices				
Vériter S. et al., 2015 [55]	hASCs	Human demineralized bone matrix (DBM)	Clinical case series of 11 patients with bone nonunions	No serious adverse events or oncological recurrences (follow up of 54 months). Fully integrated grafts. Healing of the bone nonunions
Ko E. et al., 2016 [56]	hASCs	Decellularized bovine tendon	In vitro+murine calvarial CSD	Increased osteogenic differentiation and closure of 98% of the defect with hASCs+scaffold
Zhang C. et al., 2017 [57]	hASCs	ECM+porcine small intestine submucosa (SIS)	In vitro+murine calvarial CSD	ECM-SIS plus hADSCs had the best performance vs. scaffolds alone and vs. hADSCs seeded on SIS-only scaffolds
Liu J. et al., 2018 [58]	undiff hASCs vs. osteo hASCs	Deproteinized bone matrix from rabbits (HDB)	Murine 4 mm-long radial bone defect	At 4 and 8 w both undiff hASC+HDB and osteo hASCs+HDB strong osteogenic ability. osteo hASCs+HDB practically indistinguishable from the host bone tissue
Guerrero J. et al., 2018 [59]	hASCs	Decellularized human adipose tissue (Adiscaf) vs. collagen scaffold (Ultrafoam)	Chondrogenic differentiation followed by ectopic implantation in mice	After 8 w Adiscaf produced higher amount of mineralized tissue compared to Ultrafoam. The ectopic bone formed through endochondral ossification
Wagner J.M. et al., 2019 [60]	hASCs	Human cancellous bone	In vitro+murine femur CSD	hASC+scaffold higher formation of vital bone in comparison to unseeded controls after 4 w
Calcium ceramics				
Canciani E. et al., 2016 [61]	hASCs	HA/TCP	In vitro in osteogenic conditions	The scaffold was able to enhance the osteogenic differentiation of hASCs, more than doubling the cellular alkaline phosphatase activity
Farré-Guasch E. et al., 2018 [62]	hASCs	*β*-TCP or BCP	10 patients undergoing maxillary sinus floor elevation	Seeded scaffolds had an increased vascularization of the implanted area, which ultimately determined an enhanced bone formation compared to unseeded controls
Zhang H. et al., 2018 [63]	Rabbit ASCs in a double cell sheet (DCS) with vascular and osteogenic committed ASCs	cHA	Ectopic ossification in nude mice	The DCS-cHA complexes had, better bone maturation and vascularization of the graft compared to DCS or cHA alone
Chandran S. et al., 2018 [64]	Sheep ASCs	Strontium (Sr) HA	In vitro+sheep model of osteoporosis	ASCs acted synergically with Sr ions. Enhanced osteogenic capacity of the cellular SrHA scaffold vs. acellular scaffold controls. In vivo osteointegration of the construct was superior to controls
Synthetic polymers and hybrid scaffolds				
Carvalho P.P. et al. 2014 [65]	hASCs	Wet-spun starch + PCL (SPCL)	In vitro+murine calvarial CSD	ASCs improved the osteogenic function of SPCL and promoted better bone deposition in the CSD. SPCL was able to induce osteogenic differentiation in ASCs even without osteogenic factors
Mellor L.F. et al., 2015 [66]	hASCs	Stacked nanofibrous PLA+0% or 20% of TCP nanoparticles	In vitro	In chondrogenic differentiation medium, ASCs' commitment either toward osteogenesis or chondrogenesis, depending on different calcium concentrations
Lee J. W. et al., 2017 [67]	Canine ASCs	3D-printed PCL/TCP scaffold	In vitro+canine model of a maxillary bone defect	The scaffold enhanced the osteogenic capacity of ASC process of ossification of the defect after 12 weeks, confirmed by the3D CT and histological analysis
Duan W. et al., 2018 [68]	Equine ASCs	TCP/HA (40 : 60), PEG/PLLA (60 : 40), or PEG/PLLA/TCP/HA (36 : 24 : 24 : 16)	In vitro+murine ectopic ossification model	TCP/HA and PEG/PLLA/TCP/HA promoted osteogenic differentiation of ASCs in the absence of differentiating factors. Scaffold with ASCs more ECM and osteoid tissue vs. scaffolds without cells
Natural polymers				
Correia C. et al., 2012 [69]	hASCs	Porous HFIP(hexafluoro-2-propanol)-derived silk fibroin scaffold	In vitro	The osteogenic performance at week 2 and new calcium deposition at week 7 of ASCs on silk scaffold were comparable to those of ASCs on decellularized trabecular bone
Calabrese G. et al., 2016 [70]	hASCs	Collagen/HA	In vitro	Undifferentiated ASCs on the scaffold underwent full differentiation into mature osteoblasts even without osteogenic medium
Mazzoni E. et al., 2017 [71]	hASCs	Collagen/HA	In vitro	Collagen/HA upregulated osteogenic genes and improved cellular viability and matrix mineralization, similar to osteogenic culture conditions
Toosi S. et al., 2019 [72]	Rabbit ASCs	Collagen sponge/PGA	In vitro+rabbit calvarial CSD	The scaffold promoted the healing of the defect. No difference between the scaffold-only group vs. the scaffold+ASC group.
Ko E. et al., 2017 [73]	hASCs and hASCs transfected with TAZ gene	Electrospun silk fibroin nanofiber scaffold functionalized with two-stage HA particles	In vitro+murine calvarial CSD	Constructs seeded with TAZ-transfected ASCs had the best osteogenic performance. All scaffolds seeded with hASCs proved to be superior to the unseeded scaffold

List of abbreviations: w = weeks; hASCs = human adipose stem cells; ECM = extracellular matrix; CSD = critical-sized defects; undiff hASCs = undifferentiated human adipose stem cells; osteo hASCs = osteogenically differentiated human adipose stem cells; HA = hydroxyapatite; TCP = tricalcium phosphate; *β*-TCP = *β*-tricalcium phosphate; BCP = biphasic calcium phosphate; cHA = coralline-derived hydroxyapatite; PGA = polyglycolic acid; PCL = polycaprolactone; PLA = polylactic acid; PLLA = poly-L-lactic-acid; PEG = polyethylene glycol.

**Table 2:** Novel bioactive factors for ASC osteogenic differentiation.

Authors and year of publication	Type of cells	Bioactive factor used	Vectors	Delivery system	Experimental model	Results
Lin C.Y. et al., 2013 [161]	Rabbit hASCs	Overexpression of BMP-2	Baculovirus	Gelatin	In vitro+ rabbit calvarial CSD	Increased osteogenesis of transfected ASCs. 86% of the CSD closed at 12 w
Li W. et al., 2018 [140]	osteo hASCs and bmMSCs	exosomes from osteo hASCs	Not used	PLGA/PDA matrix	In vitro+ murine calvarial CSD	Exosomes promoted osteogenic diff of bmMSCs without other diff agents. PLGA/PDA+exosomes promoted MSC migration, homing, and new bone formation, in vivo
Lu Z. et al., 2017 [169]	undiff hASCs, osteo hASCs, and HOB	Exosomes from osteo hASCs	Not used	Standard culture medium	In vitro	Exosomes of osteo hASC stimulated HOBs toward differentiation and bone formation, whereas exosomes from undiff hASCs did not
Yang S. et al., 2019 [172]	undiff hASCs and osteo hASCs	Exosomes from osteo hASCs	Not used	Standard culture medium	In vitro	Exosomes from osteo hASCs promoted osteogenic differentiation in undiff ASCs. Effects probably mediated by miR-130a-3p
Liao Y.H. et al., 2014 [177]	hASCs	miR-148b and BMP2	Baculovirus	Gelatin-coated PLGA	In vitro+murine calvarial CSD	Osteogenic differentiation of undiff ASCs was increased. Closure of the CSD in 12 w
Wang Z. et al., 2015 [180]	rat ASCs	miR-26a	Lentivirus	HA scaffold	In vitro+rat tibial CSD	Upregulation of proosteogenic genes and an increased bone-forming capacity. CSD closure in 12 weeks
Li K.C. et al., 2016 [179]	hASCs	miR-148b and BMP2	Baculovirus	Gelatin-coated PLGA	In vitro+murine calvarial CSD	Osteogenic differentiation of undiff ASCs was increased. Closure of the CSD in 12 w
Qureshi A.T. et al., 2015 [178]	hASCs	miR-148b	Photoactivated miRNA-SNP conjugates	PCL	In vitro+ murine calvarial CSD	At 12 w, transfected hASCs+PCL statistically significant better closure of a CSD vs. controls
Deng Y. et al., 2013 [181]	Rat ASCs	Anti miR-31	Lentivirus	*β*-TCP scaffold	In vitro+rat calvarial CSD	At 8 w, 35.42 ± 6.12% increase in bone volume. Better repair of CSD
Xie Q. et al., 2017 [175]	Rat ASCs	Anti miR-146	Lentivirus	Poly(sebacoyl diglyceride) (PSeD)	In vitro+rat calvarial CSD	49.8 ± 5.49% increase in bone volume

List of abbreviations: ASCs = adipose stem cells; w = weeks; hASCs = human adipose stem cells; CSD = critical-sized defects; undiff hASCs = undifferentiated human adipose stem cells; osteo hASCs = osteogenically differentiated human adipose stem cells; HA = hydroxyapatite; TCP = tricalcium phosphate; *β*-TCP = *β*-tricalcium phosphate; PCL = polycaprolactone; PLGA = poly-lactic acid-co-glycolic acid.
